# Survival outcomes for first-line antiretroviral therapy in India’s ART program

**DOI:** 10.1186/s12879-016-1887-2

**Published:** 2016-10-11

**Authors:** Rakhi Dandona, Bharat B. Rewari, G. Anil Kumar, Sukarma Tanwar, S. G. Prem Kumar, Venkata S. Vishnumolakala, Herbert C. Duber, Emmanuela Gakidou, Lalit Dandona

**Affiliations:** 1 Public Health Foundation of India, New Delhi, India; 2 Department of AIDS Control, Ministry of Health and Family Welfare, Government of India, New Delhi, India; 3 World Health Organization Country Office for India, New Delhi, India; 4 Institute for Health Metrics and Evaluation, University of Washington, Seattle, Washington USA

**Keywords:** AIDS, ART, HIV, India, Mortality, Survival

## Abstract

**Background:**

Little is known about survival outcomes of HIV patients on first-line antiretroviral therapy (ART) on a large-scale in India, or facility level factors that influence patient survival to guide further improvements in the ART program in India. We examined factors at the facility level in addition to patient factors that influence survival of adult HIV patients on ART in the publicly-funded ART program in a high- and a low-HIV prevalence state.

**Methods:**

Retrospective chart review in public sector ART facilities in the combined states of Andhra Pradesh and Telangana (APT) before these were split in 2014 and in Rajasthan (RAJ), the high- and a low-HIV prevalence states, respectively. Records of adults initiating ART between 2007-12 and 2008-13 in APT and RAJ, respectively, were reviewed and facility-level information collected at all ART centres and a sample of link ART centres. Survival probability was estimated using Kaplan-Meier method, and determinants of mortality explored with facility and patient-level factors using Cox proportional hazard model.

**Results:**

Based on data from 6581 patients, the survival probability of ART at 60 months was 76.3 % (95 % CI 73.0–79.2) in APT and 78.3 % (74.4–81.7) in RAJ. The facilities with cumulative ART patient load above the state average had lower mortality in APT (Hazard ratio [HR] 0.74, 0.57–0.95) but higher in RAJ (HR 1.37, 1.01–1.87). Facilities with higher proportion of lost to follow-up patients in APT had higher mortality (HR 1.47, 1.06–2.05), as did those with higher ART to pre-ART patient ratio in RAJ (HR 1.62, 1.14–2.29). In both states, there was higher hazard for mortality in patients with CD4 count 100 cells/mm^3^ or less at ART initiation, males, and in patients with TB co-infection.

**Conclusions:**

These data from the majority of facilities in a high- and a low-HIV burden state of India over 5 years reveal reasonable and similar survival outcomes in the two states. The facilities with higher ART load in the longer established ART program in APT had better survival, but facilities with a higher ART load and a higher ratio of ART to pre-ART patients in the less experienced ART program in RAJ had poorer survival. These findings have important implications for India’s ART program planning as it expands further.

## Background

Over the last decade, the HIV program in India has been scaled up substantially to reduce mortality and morbidity from HIV/AIDS and to improve the quality of life of those infected by HIV. The rapid scale-up of antiretroviral treatment (ART) services in recent years has improved access to ART with provision of free ART under the National AIDS Control Program (NACP- IV) [1].

The HIV program in India aims for evidence-based planning for further ART roll-out and performance monitoring [2]. However, there is a paucity of literature on survival outcomes of HIV patients on ART on a large-scale in India. The available reports are based on small samples of HIV patients, data limited to a single treatment centre, survival outcomes with TB as comorbidity, or have explored only the individual level predictors for survival on ART [3–7]. At the time of designing the study in 2012-13, our aim was to document survival outcomes and analyse the individual level and facility level predictors of survival for HIV patients on first-line ART in NACP-IV in two Indian states - Andhra Pradesh and Rajasthan. Andhra Pradesh in south India had a population of about 85 million population at that time, and the highest number of persons with HIV among any Indian state with a long-standing ART program. On the other hand, Rajasthan in north India had a population of about 70 million, and a relatively lower HIV burden with a more recent ART program [8]. Heterosexual mode of transmission is the major HIV infection route in both states [8].

## Methods

After data collection was completed in 2013, the state of Telangana was carved out of Andhra Pradesh state in June 2014. As data were collected prior to this split, we report findings for the undivided Andhra Pradesh that includes the current Andhra Pradesh and Telangana (APT) and for Rajasthan (RAJ).

Ethics approval for this study was obtained from Ethics Committees of the Public Health Foundation of India, New Delhi and the University of Washington, Seattle, USA. The study was approved by the Indian Council for Medical Research, Health Ministry Steering Committee, the Government of India and by the National AIDS Control Organization of India.

### Sample of ART facilities

In India, ART services are provided by ART centres and Link ART centres (LAC). ART centre provides pre-ART and ART services to HIV infected people, and LAC is an extension of ART centre which were established to minimize the travel need and related costs for ART patients to receive basic ART services [8]. Patients on ART are registered with ART centre to start treatment and once stable are then transferred to LAC to receive medications on a routine basis. In this study, for APT all 45 ART centres and one LAC for each ART functional in 2012 were randomly sampled. A total of 41 LACs were subsequently sampled as four ART centres in the state capital Hyderabad had no LAC. In RAJ all 16 ART centres and all 27 link ART centres functional in 2012 were sampled, of which 10 LAC were newly established, and hence data were not available for these for this study.

### Patient clinical record data

The ART patient’s clinical record (known as *white card*) is maintained at the facilities that provide ART services. It is used to document demographic information of the patient; risk factors; and various treatment and clinical details, and follow-up details for each visit. We aimed to sample 75 and 35 adult patient records at each ART centre and LAC, respectively in APT, for the last five financial years at the time of starting data collection (April 2007 to March 2012). For RAJ, the aimed sample was 180 and 30 adult patient records at each ART centre and LAC, respectively, during the last 5 financial years at the time of starting data collection (April 2008 to March 2013). With this approach, at least 3000 adult patients on ART were finally expected to be part of the study in each state. Only records of patients who were initiated on first line ART between 6 and 60 months prior to the date of data collection were considered eligible. We used the ART enrolment register which includes documentation of the ART initiation date for each patient to arrive at the total number of eligible adult patient records at each facility. We then used a pre-defined sampling strategy to sample the eligible patient records at each ART facility - the total number of adult patients who had initiated ART within the inclusion time period was divided by the required number of adult patient records to be sampled to arrive at the sampling interval. A random number was picked within this sampling interval to select the first record, and then the records were sampled systematically using the sampling interval until the desired sample was achieved.

### Data collection procedure

Data were collected electronically using Datstat Illume Survey Manager 5.1 (DatStat Inc., Seattle, WA). The program used for capturing data was a replica of the white card. Data extractors trained in study procedures recorded data “as is” from the white cards to reflect the actual data in each white card. The current status of the patient (alive, dead, lost to follow-up, or transferred to another facility) was recorded from the ART enrolment register. Ten percent or more of data collected were checked daily by the field team supervisor to ensure the quality of data collected. Formal consent of the senior-most person responsible at each ART facility was obtained to collect these data. Data were collected in APT from February to May 2013 and in RAJ during June-July 2013.

### Data analysis

#### Survival probability

The probability of survival of HIV patients on ART at 12 and 60 months was estimated using the Kaplan-Meier product limit estimation method. The duration of survival was calculated from the month of ART initiation to the month of death or to the last visit for the alive patients. Censoring based on the last date of visit to the ART was done for patients who were lost to follow-up (LFU) or transferred out of the facility. We report overall survival probability of HIV patients on ART at 12 and 60 month for the two sexes and by baseline CD4 cell count. As mortality at the facility level is mostly not captured among HIV patients who were LFU, we adjusted the survival with mortality among LFU patients as shown in the equation below that has been previously used in the literature: [9]

$$ \mathrm{S}\left(\mathrm{t}\right) = 1 - \left[\mathrm{ML}\left(\mathrm{t}\right) + \mathrm{L}\left(\mathrm{t}\right)*\mathrm{M}\mathrm{N}\mathrm{L}\left(\mathrm{t}\right)\right] $$

where S(t) = adjusted ART survival in last 5 years; L(t) = Proportion of LFU patients in 5 years; ML(t) = Mortality estimated in LFU patients; and MNL(t) = Mortality observed in patients in care (1- predicted Kaplan-Meier survival estimates).

As an inverse relation between mortality among LFU patients and the rate of LFU in the ART program has been previously reported from an analysis of several HIV programs in Africa, we used the following equation from this analysis to estimate mortality among LFU patients in each facility in APT and RAJ: [9]

$$ {\mathrm{ML}}_{\mathrm{i}}= \exp\ \left(\mathrm{a} + {\mathrm{br}}_{\mathrm{i}}\right)/\left(1 + \exp\ \left(\mathrm{a} + {\mathrm{br}}_{\mathrm{i}}\right)\right) $$

where ML_i_ = mortality among non-LFU patients in each facility, *r* = proportion of LFU patients in each facility, a = 0.57287 and b = −4.04409.

Finally, we calculated the weighted average of ML for each state by using the total patients on ART in each facility in each state. The weighted average of estimated mortality among LFU patients was 0.43 for APT and 0.61 for RAJ at 5 years. We considered 20 % lower and higher levels than the point estimates for ML(t) for sensitivity analysis for the probabilities of ART survival, which was performed using Monte Carlo simulations by doing 100,000 iterations with the @Risk software (Palisade UK Europe Ltd). We used random values between these plausible ranges to obtain the 95 % uncertainty interval (UI) for the probabilities of survival estimates. We report survival probability at 60 months that is adjusted for LFU mortality.

#### Predictors of mortality

Cox proportional hazard model was used to determine the predictors of mortality with select patient demography and clinical indicators (ART regimen, CD4 count at start of ART, co-existing tuberculosis, history of alcohol use). We also included facility related variables - cumulative ART patient load at ART centre, ratio of cumulative ART patients to pre-ART patients, and percent of cumulative LFU patients. The cumulative data over the 5-year study period was used to calculate the average value for each of these variables per state. Facilities were categorised as having below/equal or above the average value for cumulative ART patient load and ratio of cumulative ART patients to pre-ART patients; and were categorised into three equal groups based on the percent of cumulative LFU patients for the analysis. For this analysis, the ART patients undergoing treatment at LAC were considered together with the parent ART centre which was their primary registration for ART.

The 95 % confidence intervals (CI) are reported where relevant. The median CD4 count at the ART initiation is presented for the alive, dead and LFU patients separately and range is also reported. Log rank test was used to examine the test of significance for survival probability. Data from 82 to 41 facilities were available for analysis in APT and RAJ, respectively. Data were analysed using the statistical software STATA (version 13.1, StatCorp, USA).

## Results

A total of 3340 adult patient records were extracted for analysis in APT state of which 3280 (98.2 %) records had information available on the current status of the patient at the time of data collection. Among these, 2130 (64.9 %) were alive and on ART, 437 (13.3 %) had died, 432 (13.2 %) were LFU, and 281 (8.6 %) were transferred out to another ART facility during the study period. In RAJ state, 3241 adult patient records were extracted for analysis of which 3198 (98.7 %) had information available on the current status of the patient at the time of data collection. Among these, 2554 (79.9 %) were alive and on ART, 393 (12.3 %) were dead, 115 (3.6 %) were LFU, and 136 (4.3 %) were transferred out to another ART facility.

**Fig. 1:**
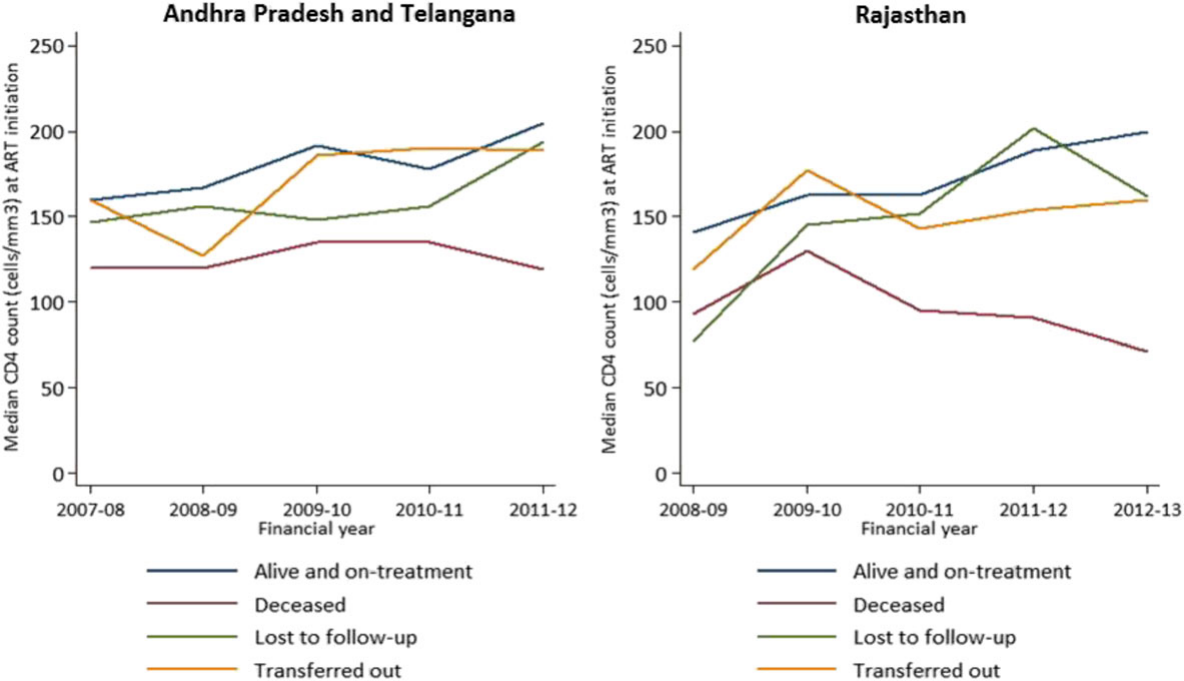
Yearly trends in baseline median CD4 cell counts for HIV patients on ART in Andhra Pradesh and Telangana (2007-12) and in Rajasthan (2008-13) based on current status of the patient

The demographic and clinical characteristics of adult patients on ART are summarized in Table 1. The median age of patients on ART was 35 years in both states (Interquartile range, IQR 29–40 years). The median CD4 cell count at ART initiation was 172 cells/mm3 (IQR 104–236) in APT and 159 cells/mm3 (IQR 86–240) in RAJ. Significantly more patients had CD4 count of < =100 cells/mm3 at ART initiation in RAJ (29.4 %) than in APT (23.4 %; *p* < 0.001). The patients who were alive and on treatment showed an increasing trend in median CD4 cell counts at ART initiation over the years in both the states (Fig. 1). The overall baseline median CD4 cell count of deceased patients [126 cells/mm3 (IQR 66–194) in APT and 93 cells/mm3 (IQR 48–159) in RAJ) were comparatively lower than the patients who were alive and on treatment in both the states [184 cells/mm3 (IQR 115–245) in APT and 172 cells/mm3 (IQR 98–247) in RAJ].

**Table 1:** Demographic and clinical characteristics of patients on ART, and facility-related indicators in Andhra Pradesh and Telangana (2007-12) and in Rajasthan (2008-13)

	Andhra Pradesh and Telangana (*N* = 3,340)^a^		Rajasthan (*N* = 3,241)^b^	
Variables	Total (% of N)	Deceased (%)	Total (% of N)	Deceased (%)
*Patient level*				
*Year of ART initiation* ^c^				
2007-08	365 (10.9)	58 (15.9)		
2008-09	458 (13.7)	68 (14.9)	422 (13.0)	72 (17.1)
2009-10	767 (23.0)	97 (12.7)	651 (20.1)	67 (10.3)
2010-11	829 (24.8)	124 (15.0)	749 (23.1)	90 (12.0)
2011-12	919 (27.5)	90 (9.8)	850 (26.2)	85 (10.0)
2012-13			569 (17.6)	79 (13.9)
*Sex of patient*				
Male	1,724 (51.6)	273 (15.8)	1,860 (57.4)	285 (15.3)
Female	1,561 (46.7)	159 (10.2)	1,360 (42.0)	108 (7.9)
*Age of patients (years)*				
16–29	857 (25.7)	98 (11.4)	720 (22.2)	66 (9.2)
30–49	2,152 (64.4)	287 (13.3)	2,250 (69.4)	278 (12.4)
= >50	329 (9.9)	52 (15.8)	271 (8.4)	49 (18.1)
*CD4 count at ART initiation (cells/mm3)*				
< =100	782 (23.4)	168 (21.5)	954 (29.4)	209 (21.9)
101–250	1,870 (56.0)	226 (12.1)	1,562 (48.2)	150 (9.6)
>250	659 (19.7)	41 (6.2)	696 (21.5)	31 (4.5)
*Co-existing tuberculosis*				
No	2,823 (84.5)	336 (11.9)	2,582 (79.7)	276 (10.7)
Yes	515 (15.4)	101 (19.6)	627 (19.4)	117 (18.7)
*Ever use of alcohol*				
No	2,739 (82.0)	319 (11.7)	2,709 (83.6)	299 (11.0)
Yes	599 (17.9)	118 (19.7)	519 (16.0)	94 (18.1)
*Baseline ART regimen* ^d^				
STV based regimen with/without EFV	1,377 (41.2)	215 (15.6)	832 (25.7)	146 (17.6)
ZDV based regimen with/without EFV	1,542 (46.2)	166 (10.8)	2,131 (65.8)	205 (9.6)
*Facility level variables*				
*Cumulative ART patient load* ^e^				
Below or equal to state average	1,891 (56.6)	262 (13.9)	1,621 (50.0)	190 (11.7)
Above state average	1,449 (43.4)	175 (12.1)	1,620 (50.0)	203 (12.5)
*Ratio of cumulative ART patients to pre-ART patients* ^f^				
Below or equal to state average	1,998 (59.8)	275 (13.8)	2,433 (75.1)	271 (11.1)
Above state average	1,342 (40.2)	162 (12.1)	808 (24.9)	122 (15.1)
*Percent of cumulative loss to follow-up patients* ^g^				
Category 1	1,070 (32.0)	123 (11.5)	1,352 (41.7)	174 (12.9)
Category 2	1,090 (32.6)	148 (13.4)	1,375 (42.4)	150 (10.9)
Category 3	1,180 (35.3)	166 (14.1)	514 (15.9)	69 (13.4)

^a^Data missing for Andhra Pradesh and Telangana: year of ART initiation for 2; sex of patients for 55; age of patients for 2; CD4 count at ART initiation for 29; co-existing tuberculosis for 2; ever use of alcohol for 2; baseline ART regimen for 421

^b^Data missing for Rajasthan: sex of patients for 21; CD4 count at ART initiation for 29; co-existing tuberculosis for 32; ever use of alcohol for 13; baseline ART regimen for 278

^c^Financial year

^d^STV is Stavudine, ZDV is Zidovudine, and EFV is Efavirenz

^e^Average for Andhra Pradesh and Telangana is 4844 and 4307 for Rajasthan

^f^For Andhra Pradesh and Telangana is 0.59 and 0.70 for Rajasthan

^g^The categories for Andhra Pradesh and Telangana are <5.6 %, >5.6–8.4 % and >8.4 %; and <3.1 %, 3.1–6.5 % and >6.5 % for Rajasthan

### Survival probability

Among the 437 and 393 patients who had died in APT and RAJ, respectively, 188 (43 %) in APT and 191 (48.6 %) patients in RAJ had died within 6 months of starting ART. The unadjusted mortality rate among patients on ART in APT and RAJ was 6.83 and 7.18 per 100 patient-years at 60 months, respectively. The median survival time was 22 months in APT and 18 months in RAJ.

**Fig. 2:**
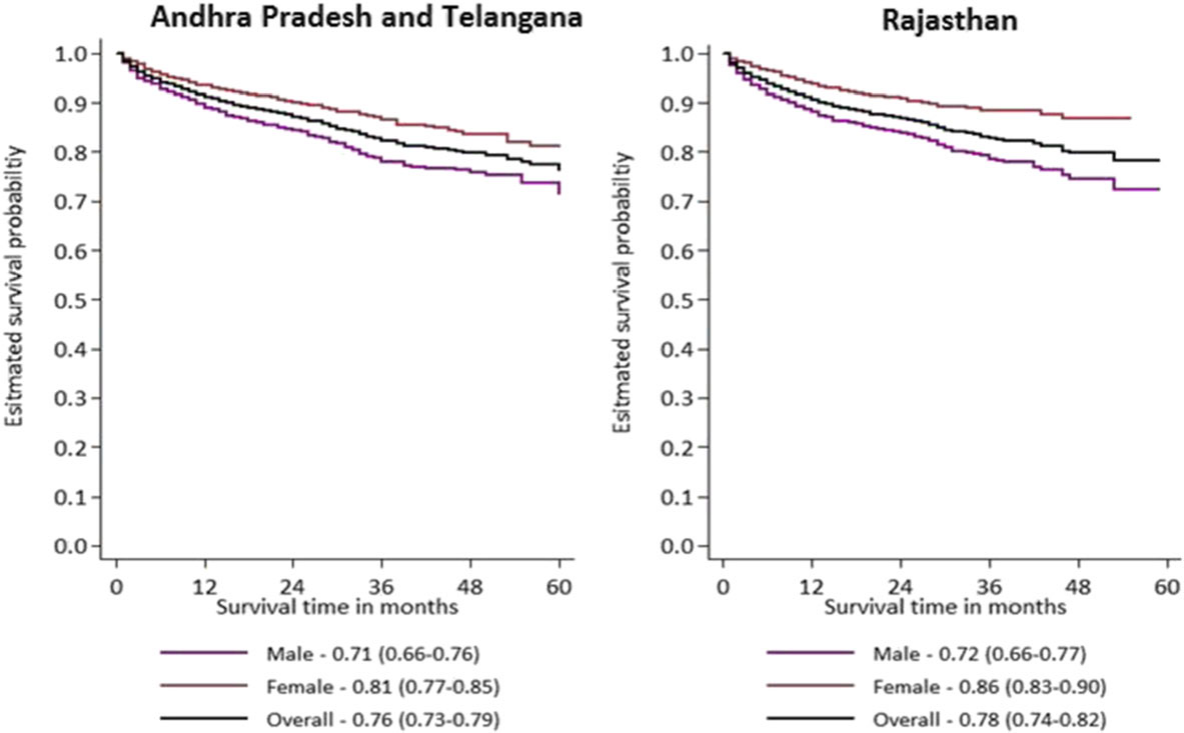
Kaplan-Meier unadjusted survival curve for HIV patients on ART for males and females in Andhra Pradesh and Telangana (2007-12) and Rajasthan (2008-13)

**Fig. 3:**
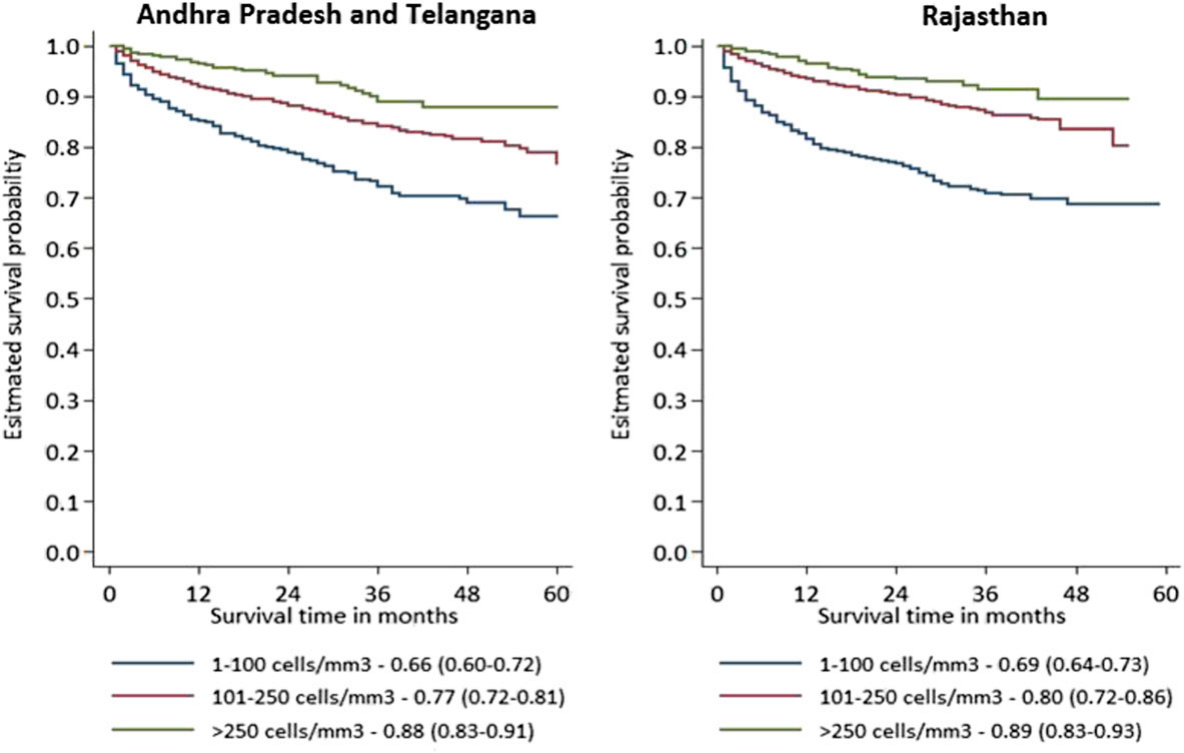
Kaplan-Meier unadjusted survival curve for HIV infected patients on ART by CD4 count at ART initiation (cells/mm^3^) in Andhra Pradesh and Telangana (2007-12) and Rajasthan (2008-13)

The estimated unadjusted survival probability on ART at 12 and 60 months was 91.2 % (95 % CI 90.1–92.1) and 76.3 % (95 % CI 73.0–79.2) in APT, respectively; and 90.6 % (95 % CI 89.4–91.6) and 78.3 % (95 % CI 74.4–81.7) in RAJ (Fig. 2). The probability of survival was higher among females than males in both states (log rank test, *p* < 0.001; Fig. 2), and was significantly lower for patients with CD4 count <101 cells/mm3 at ART initiation than those with CD4 count >250 cells/mm3 in both the states (log rank test, *p* < 0.001; Fig. 3). After adjusting for the assumed higher mortality among LFU, the adjusted survival probability on ART at 60 months was 70.6 % (95 % UI 67.0–73.8) in APT and 76.1 % (95 % UI 72.2–79.6) in RAJ.

### Predictors of mortality

Table 2 shows the results with the Cox proportional hazard model for the predictors of mortality among HIV patients on ART. The findings at the patient level for both the states were similar. Patients with CD4 count <101 cells/mm^3^ at ART initiation had a higher hazard for mortality than patients with CD4 count >250 cells/mm3 (HR 3.36, 95 % CI 2.29–4.95 in APT and HR 3.71, 95 % CI 2.47–5.58 in RAJ). Patients with history of alcohol consumption had significantly higher risk of dying than who never consumed alcohol in APT (HR 1.57, 95 % CI 1.22–2.02) and in RAJ (HR 1.42, 95 % CI 1.09–1.86). Males as compared with females, and patients with TB co-infection had a higher hazard for death. The patients on Zidovudine-based ART regimen had a lower hazard for mortality than those on the Stavudine-based ART regimen in both states.

**Table 2:** Determinants of mortality among HIV patients on ART using Cox proportional hazard model for Andhra Pradesh and Telangana (2007-12) and Rajasthan (2008-13). CI denotes confidence interval

	Andhra Pradesh and Telangana^a^	Rajasthan^b^
Variables	Hazard ratio (95 % CI)	Hazard ratio (95 % CI)
*Patient level variables*		
*Sex of patient*		
Male	1.36 (1.06–1.73)	1.83 (1.41–2.38)
Female	1.00	1.00
*Age of patients (years)*		
16–29	1.00	1.00
30–49	1.00 (0.77–1.29)	1.00 (0.75–1.34)
= >50	1.24 (0.85–1.79)	1.25 (0.84–1.88)
*CD4 count at ART initiation (cells/mm3)*		
< =100	3.36 (2.29–4.95)	3.71 (2.47–5.58)
101–250	1.94 (1.33–2.82)	1.73 (1.15–2.62)
>250	1.00	1.00
*Co-existing tuberculosis*		
No	1.00	1.00
Yes	1.42 (1.11–1.81)	1.31 (1.04–1.67)
*Ever use of alcohol*		
No	1.00	1.00
Yes	1.57 (1.22–2.02)	1.42 (1.09–1.86)
*Baseline ART regimen* ^c^		
STV based regimen with/without EFV	1.00	1.00
ZDV based regimen with/without EFV	0.67 (0.55–0.83)	0.62 (0.50–0.78)
*Facility level variables*		
*Cumulative ART patient load* ^d^		
Below or equal to state average	1.00	1.00
Above state average	0.74 (0.57–0.95)	1.37 (1.01–1.87)
*Ratio of cumulative ART patients to pre-ART patients* ^e^		
Below or equal to state average	1.00	1.00
Above state average	0.90 (0.71–1.15)	1.62 (1.14–2.29)
*Percent of cumulative loss to follow-up patients* ^f^		
Category 1	1.00	1.00
Category 2	1.16 (0.87–1.54)	1.22 (0.94–1.58)
Category 3	1.47 (1.06–2.05)	1.40 (1.00–1.95)

^a^Data missing for Andhra Pradesh and Telangana: year of ART initiation for 2; sex of patients for 55; age of patients for 2; CD4 count at ART initiation for 29; co-existing tuberculosis for 2; ever use of alcohol for 2; baseline ART regimen for 421

^b^Data missing for Rajasthan: sex of patients for 21; CD4 count at ART initiation for 29; co-existing tuberculosis for 32; ever use of alcohol for 13; baseline ART regimen for 278

^c^STV is Stavudine, ZDV is Zidovudine, and EFV is Efavirenz

^d^Average for Andhra Pradesh and Telangana is 4844 and 4307 for Rajasthan

^e^For Andhra Pradesh and Telangana is 0.59 and 0.70 for Rajasthan

^f^The categories for Andhra Pradesh and Telangana are <5.6 %, >5.6–8.4 % and >8.4 %; and <3.1 %, 3.1–6.5 % and >6.5 % for Rajasthan

At the facility level, facilities with a cumulative ART patient load above the average for the state facilities had lower mortality in APT (HR 0.74, 95 % CI 0.57–0.95) but had higher mortality in RAJ (HR 1.37, 95 % CI 1.01–1.87). The facilities in APT with proportion of LFU patients higher than the state average had significantly higher mortality (HR 1.47, 95 % CI 1.06–2.05); the trend in RAJ was similar but did not reach statistical significance. On the other hand, facilities in RAJ with higher ART to pre-ART patient ratio had a significantly higher hazard for mortality (HR 1.62, 95 % CI 1.14–2.29).

## Discussion

As public sector facilities provide ART to most patients in India, this sample of over 6500 adult patients on ART in two major states is fairly representative of a high and a low HIV burden state in India. This analysis of data covering 5 years reveals that the overall survival probability of HIV patients on ART at 60 months was reasonable at 76–78 %, and that the survival rates were similar in the high- and low-HIV burden states, with the former having a longer standing public funded ART program in place.

The survival rates in our data at 60 months are similar to those reported previously from three centres in southern India [3, 10]. Consistent with the published literature, a significant proportion of deaths occurred within the first 6 months of ART initiation [3–5, 7, 11]. Poor survival of males on ART as compared with females in our population has been documented previously from India and elsewhere [3, 6, 12–16]. Factors such as poor treatment seeking behaviour and non-adherence to treatment, and increased risk of LFU have been reported previously as possible reasons for higher mortality among males on ART [17–20]. The median CD4 count at ART initiation was lower for males than females in both the states, and 58 % of LFU in APT and 65 % in RAJ were males in our study. This finding suggests that it would be useful for the HIV services to make males more aware of the benefits of timely initiation of ART for better survival outcome. Both a low CD4 count at ART initiation and co-existing TB have been previously reported to be associated with poorer survival outcomes among Indian patients [3, 4, 17, 21–23]. The overall median CD4 count at ART initiation in this study had increased significantly over the 5 years in both APT (154 to 193) and RAJ (132 to 174). However, these data were not for those who had died. As a lower CD4 count is associated with delayed ART initiation and with higher attrition while on treatment, [17, 22] the program could focus more on ensuring adherence and follow-up of the patients with lower CD4 count to further improve the survival outcomes. With regard to TB, NACP-IV has clearly identified HIV-TB coordination including cross-referral, detection and treatment as one of the objectives in the revised strategy that aims to further the integration between HIV and TB services, [24] in particular to prevent LFU and early initiation of ART [25–27].

Inclusion of data from a large number of facilities in this study allowed assessment of facility-level variables that influence survival on ART. These findings are relevant for program planning. The ART patient load was an important predictor of mortality in both states, albeit differently. In APT, facilities with a higher load had better survival outcomes possibly because of a longer established ART program that has likely acquired more experience leading to better outcomes. However, in RAJ, facilities with higher ART patient load had poorer survival outcome, as did facilities with a higher ratio of ART to pre-ART patients. This higher patient load in the less experienced ART program in RAJ may be resulting in difficulty in handling patients, which indicates the need for strengthening facilities in RAJ with high or increasing ART load through monitoring of their human resources, supplies and infrastructure. In addition, even though both states had more pre-ART than ART patients across the facilities, the average ART to pre-ART patient ratio was relatively higher in RAJ.

The reasonable survival outcomes in the two states, which were not significantly different from each other without and with adjusting for mortality in the LFU patients, are encouraging for the national HIV program. Over the study period, the LFU proportion remained fairly consistent in APT, and was similar to that reported previously [17, 22]. Factors associated with poor patient retention have been documented for APT, [17, 22] and more effective and robust tracking of LFU is needed to improve survival outcomes. The significantly lower proportion of LFU in RAJ was a likely a result of a recent exercise carried out by the State AIDS Control Society to trace LFUs in order to bring them back to the treatment cycle. It is possible that some LFU patients may have initiated ART at another facility. However, it is not possible to track mobility of individual patients between the ART facilities in the program yet. To address this challenge, NACO is considering use of SMART cards with biometric identification for each patient which could facilitate not only tracking of patients but also potentially improve adherence and access to treatment [28].

Our study limitations include missing data, non-usable information on treatment adherence in the white cards, and survival status of transferred out and LFU patients as these were not readily available in the patient records. Despite these limitations, these large sample data collected from routine patient records are generalizable as all ART centres in both states were included. Data utilised for this study were obtained from paper forms/registers used in routine service conditions by the providers in the facilities, and thus are reflective of the ground reality.

## Conclusions

In conclusion, these data have highlighted the benefits of investment in ART in India which is associated with a reasonably good over survival rate at 5 years, and have identified important determinants of survival on ART at the facility-level in addition to patient-level factors that can inform improvement of the ART services in India. An important program-relevant message from these findings is that ART survival could potentially be improved further if facilities with higher load get specific attention in the initial phase in Indian states with a more recent ART program.

## Abbreviations

AIDS: Acquired immunodeficiency syndrome
APT: Andhra Pradesh and Telangana
ART: Antiretroviral therapy
CI: Confidence interval
HIV: Human immunodeficiency virus
HR: Hazard ratio
LAC: Link ART centre
LFU: Lost to follow-up
NACP: National AIDS Control Programme
NACO: National AIDS Control Organisation
UI: Uncertainty interval
RAJ: Rajasthan
TB: Tuberculosis
